# Regional Utilization of Preventive Services in the 55-Plus Age Group: Protocol for a Mixed Methods Study

**DOI:** 10.2196/33512

**Published:** 2022-01-12

**Authors:** Ilona Hrudey, Annemarie Minow, Svenja Walter, Stefanie March, Enno Swart, Christoph Stallmann

**Affiliations:** 1 Institute of Social Medicine and Health Systems Research Faculty of Medicine Otto-von-Guericke University Magdeburg Magdeburg Germany; 2 Department of Social Work, Health and Media Magdeburg-Stendal University of Applied Sciences Magdeburg Germany; 3 Department of Studies and Teaching Medicine University of Lübeck Lübeck Germany

**Keywords:** prevention, utilization, determinants, barriers, Germany, cancer screening, medical check-ups, vaccinations, preventive dental care, older people

## Abstract

**Background:**

In Germany, the proportion of people with chronic diseases and multimorbidity is increasing. To counteract the emergence and worsening of age-related conditions, there is a need for preventive care structures and measures. The preventive services that are financed by statutory health insurance (SHI; eg, vaccinations, cancer screening) are only used by part of the German population. There are no current findings about the utilization of these services by older adults in the eastern German federal state of Saxony-Anhalt, which is particularly strongly affected by demographic change.

**Objective:**

The aim of this study is to investigate the actual utilization and determinants of, reasons for, and barriers to utilization of preventive services financed by the SHI in Saxony-Anhalt in the 55-plus age group.

**Methods:**

In this study, a convergent mixed methods design is used. The actual use of preventive services will be shown by means of (1) a claims data analysis looking at data on statutory outpatient medical care from both the Central Research Institute of Ambulatory Health Care in Germany (Zi) and the Association of Statutory Health Insurance Dentists in Saxony-Anhalt (KZV LSA). The determinants, attitudes, and behaviors associated with use will be analyzed through (2) a cross-sectional survey as well as (3) qualitative data from semistructured interviews with residents of Saxony-Anhalt and from focus group discussions with physicians. (4) A stock take and systematic evaluation of digitally available informational material on colorectal cancer screening, by way of example, provides an insight into the information available as well as its quality. The conceptual framework of the study is the behavioral model of health services use by Andersen et al (last modified in 2014).

**Results:**

(1) The Zi and KZV LSA are currently preparing the requested claims data. (2) The survey was carried out from April 2021 to June 2021 in 2 urban and 2 rural municipalities (encompassing a small town and surrounding area) in Saxony-Anhalt. In total, 3665 people were contacted, with a response rate of 25.84% (n=954). (3) For the semistructured interviews, 18 participants from the 4 different study regions were recruited in the same period. A total of 4 general practitioners and 3 medical specialists participated in 2 focus group discussions. (4) For the systematic evaluation of existing informational material on colorectal cancer screening, 37 different informational materials were identified on the websites of 16 health care actors.

**Conclusions:**

This study will provide current and reliable data on the use of preventive services in the 55-plus age group in Saxony-Anhalt. It will yield insights into the determinants, reasons, and barriers associated with their utilization. The results will reveal the potential for preventive measures and enable concrete recommendations for action for the target population of the study.

**Trial Registration:**

German Clinical Trials Register DRKS00024059; https://www.drks.de/drks_web/navigate.do?navigationId=trial.HTML&TRIAL_ID=DRKS00024059

**International Registered Report Identifier (IRRID):**

DERR1-10.2196/33512

## Introduction

### Background

The eastern German federal state Saxony-Anhalt (part of the former German Democratic Republic) is strongly affected by demographic change. In 2019, the proportion of the population of Saxony-Anhalt aged 65 years and older was approximately 27% of the total population [1]. The aging of the population is accompanied by an increasing need for care, which is leading to growing structural challenges in medical care. For instance, the density of physicians in Saxony-Anhalt in 2020 was 197.9 physicians per 100,000 inhabitants. In comparison, the density of physicians in the western German federal state of Bavaria was 221.5 physicians per 100,000 inhabitants [2]. According to the German Index of Socioeconomic Deprivation, developed by the Robert Koch Institute, Saxony-Anhalt has a comparatively high level of socioeconomic deprivation. Empirical evidence has shown that this is associated with negative health impacts such as an accumulation of health risks. Moreover, the prevalence of individual risk factors (eg, smoking, obesity) is higher in Saxony-Anhalt than the national average [3].

As in many developed countries, in Germany, the proportion of people with chronic diseases and multimorbidity is rising due to increasing life expectancy and advances in medical technology [4]. Furthermore, from 1959 to 1968, there were very high birth rates in East Germany. The children born in this period, the so-called “baby boomers,” currently constitute the largest age group in Germany [5]. The first baby boomers will leave the labor force in 2025 and pass the aforementioned threshold of 65 years of age. It is expected that there will be an increase in chronic diseases in this age group in the coming years [6]. Cardiovascular diseases and cancer, which can be influenced by preventive measures, mainly cause the burden of disease in Germany [4]. According to hospital diagnosis–related group statistics, cardiovascular diseases caused 8.8% of all full inpatient hospital cases in 2019. In Saxony-Anhalt, the full inpatient hospitalization rate is 17.4% higher than the national average [7].

Preventive care structures and measures are needed to counteract the development and deterioration of age-associated diseases and to maintain and strengthen health, independence, and participation in social life into old age. Therefore, in addition to the avoidance of disease risks, prevention in old age pursues the goal of strengthening physical, psychological, and social resources, also in the case of existing health-related and functional restrictions [8]. In this context, primary and secondary preventive measures are particularly relevant. In Germany, public health is the responsibility of the federal states and covers, for example, the surveillance of infectious diseases. Some public health services, such as medical and dental check-ups, vaccinations, and cancer screening are financed by statutory health insurance (SHI), which insures approximately 87% of the German population. In Germany, all citizens must have either statutory or private health insurance, whereby a number of criteria regulates who is insured in which system [9]. Further details about the German health system and its financing were published in [9,10]. Details on the scope, eligible populations, and examination intervals of SHI-financed preventive services can be found in [11,12]. In 2015, the “Act to Strengthen Health Promotion and Disease Prevention” (“Gesetz zur Stärkung der Gesundheitsförderung und der Prävention”) was passed by the German legislature. It aims particularly to strengthen health promotion in living environments (settings). Moreover, it contains regulations to strengthen the vaccination system and to further develop health and early detection examinations.

The effectiveness of preventive services depends on the extent of their use. In Germany, preventive services have been underutilized. In 2018, only about 35% of the German population aged 65 years and older had been vaccinated against seasonal influenza. This is below the European Union average of 44% [4]. In comparison, in 2018/2019, the vaccination coverage rate for seasonal influenza in Saxony-Anhalt was 59% among people aged 60 years and older, which is above the national average [13]. The target vaccination coverage rate defined by the World Health Organization for older people is at least 75%. This is not nearly achieved, neither nationwide nor in Saxony-Anhalt. The cancer screening tests offered (eg, breast cancer screening) are only used by part of the eligible population, but there is a mixed picture in terms of utilization rates depending on the screening program [4]. It should be noted that cancer screening can also cause harm (eg, overdiagnosis, false-positive results). Therefore, since 2013, Germany's health policy aims to increase informed decision making for or against screening [14]. Several studies have shown that numerous determinants and barriers influence the utilization of preventive services in Germany. The level of utilization differs according to social status, sex, age, residential region (eastern and western Germany), health status, and health-related behavior, among other factors [15-18].

Based on the initial situation described in the previous paragraphs, it can be assumed that, in the federal state Saxony-Anhalt, there are comparatively pronounced determinants that are negatively associated with the utilization of preventive services. However, for Saxony-Anhalt’s older population, there are no current, representative findings on the utilization of these services and the associated influencing factors.

### Objectives of the Study

In the mixed methods study, “Prevention in old-age Saxony-Anhalt” (“Prävention im Alter Sachsen-Anhalt” [PrimA LSA]), we examine the actual utilization and the determinants, reasons, and barriers influencing the use of preventive services in Saxony-Anhalt in the 55-plus age group. The aim is to identify further potential for prevention in the aging population and to derive recommendations for measures leading to needs-driven improvement or further development of preventive services and their utilization. Our focus is on medical services for primary prevention (vaccinations) and secondary prevention (cancer screening, medical and dental check-ups).

## Methods

### Overview of the Study Design

**Figure 1:**
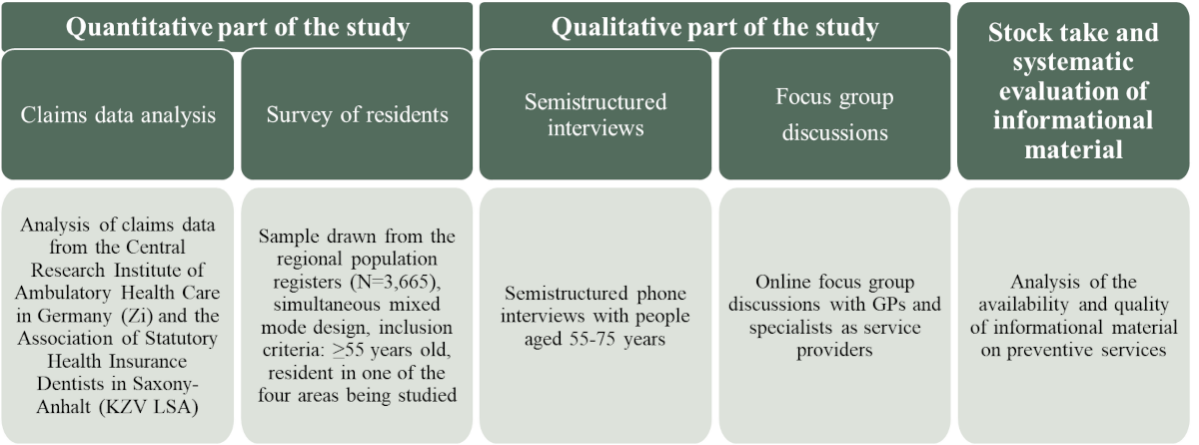
Overview of the study design. GP: general practitioner.

The target population of the study consists of residents aged 55 years and older living in the eastern German federal state of Saxony-Anhalt. By means of an analysis of claims data—the billing data from the SHI—the actual utilization of preventive services in the target population will be shown. The determinants, attitudes, and behaviors associated with utilization will be analyzed using a survey as well as qualitative data from semistructured interviews with residents of Saxony-Anhalt and from focus group discussions with physicians. A search for and systematic evaluation of existing informational material on the applicable preventive services provide insight into the information available and its quality. In addition, it enables further development of existing informational material and an analysis of the relevant health care actors. Figure 1 provides an overview of the study design and its methodological strands.

**Figure 2:**
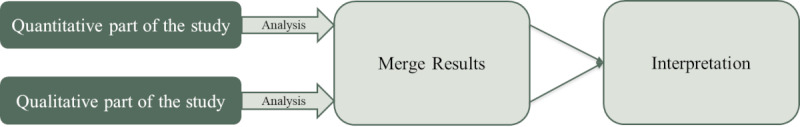
Flowchart of the convergent mixed methods design of the study. Modified from [19].

In this study, a convergent mixed methods design is used (Figure 2). The qualitative and quantitative data are collected simultaneously with equal priority. First, the data will be analyzed separately and will then be merged and interpreted together. This ensures a comprehensive and complementary understanding of the investigated research topic [19].

Our study is accompanied and supported by regional cooperation partners, like nonstatutory welfare institutions, the Association of Statutory Health Insurance Physicians in Saxony-Anhalt (Kassenärztliche Vereinigung Sachsen-Anhalt [KVSA]) and the Association of Statutory Health Insurance Dentists in Saxony-Anhalt (Kassenzahnärztliche Vereinigung Sachsen-Anhalt [KZV LSA]). In regular meetings, we discuss the current state of the study as well as further steps in order to ensure the transfer of research to practice. In addition, the study is part of the academic training of specialists in the field of prevention and health promotion.

The conceptual framework of the study is the behavioral model of health services use by Andersen et al (last modified in 2014) [20], which enables the identification of factors influencing the extent to which preventive services are utilized. It encompasses individual and contextual characteristics, the health behavior of individuals, as well as the outcomes of the use of health services. At the level of individual and contextual characteristics, the model distinguishes predisposing (eg, sex, age), enabling (eg, health insurance status, accessibility of health care facilities), and need factors (eg, existing risk factors) [20]. In line with Andersen et al [20] and other previous studies that examined the determinants, reasons, and barriers to the use of preventive services [15-18], we assume that the utilization depends especially on the aforementioned 3 factors. The results of the qualitative and quantitative parts of the study will be analyzed and related to each other based on the dimensions of the model by Andersen et al [20].

### Claims Data Analysis

Current and reliable data on the actual use of preventive services are indispensable for target group–specific communication about these services as well as for the evaluation and further development of existing recommendations and programs (eg, organized cancer screening programs). For this reason, aggregated claims data from statutory outpatient health care from the Central Research Institute for Ambulatory Health Care in Germany (Zi) and the KVSA will be analyzed in the study. These data provide information about services billed to the SHI for all people aged 55 years and older who are insured under the SHI scheme in the federal state of Saxony-Anhalt. For legal or technical reasons, both institutions keep the data for different periods.

The Zi has national statutory health care billing and prescription data from outpatient care that are submitted by all 17 associations of SHI physicians in Germany [21]. Within the framework of our analysis, we will consider the following groups of services: consultations and examinations for early cancer detection, medical check-ups as well as the vaccinations recommended by the German Standing Committee on Vaccination (STIKO) for the relevant age group (eg, influenza, streptococcus pneumoniae, herpes zoster) [22]. The utilization rates will be calculated as the number of insured people who have made use of a certain service at least once within a defined reporting period, divided by the population eligible for the respective service among those with SHI. The key epidemiological figures will be shown for the entire target population and stratified according to age group (5-year age brackets up to age 95 years and older), sex, region, and reporting year as well as other characteristics, for example the existence of chronic diseases or diseases with a vaccination indication (eg, cardiovascular diseases, diabetes) [22]. The utilization will be presented chronologically from 2011 to 2020. Since the Zi has national claims data at its disposal, the utilization rate in Saxony-Anhalt will be compared with the rate for the entire German federal territory. Regional differences in Saxony-Anhalt will be examined at the district and urban district levels (German: *Landkreis* and *kreisfreie Stadt*, respectively).

In order to obtain additional empirical information about the preventive dental care of older residents in Saxony-Anhalt, data from the KZV LSA will be analyzed. Here, we will consider the utilization of dental check-ups, the costs of which are covered by the SHI once in every calendar half year, as well as visits to provide dentistry services to SHI-insured persons in need of inpatient long-term care. The calculation of the utilization rates will be carried out the same way as with the Zi data. The key figures will be calculated for the entire target population as well as stratified according to age group (5-year age brackets up to age 95 years and older), sex, and reporting year. The utilization from 2016 to 2020 will be presented chronologically. When implementing the described claims data analysis, we will follow the guidelines of Good Practice of Secondary Data Analysis [23].

### Survey of Residents

The determinants, reasons, and barriers influencing the utilization of preventive services will be elicited using a cross-sectional survey. We selected 2 urban and 2 rural municipalities in Saxony-Anhalt as study areas: Magdeburg, Halle (each with around 240,000 inhabitants), Wanzleben-Börde (approximately 14,170 inhabitants), and Sangerhausen (about 26,200 inhabitants). The 4 municipalities differ in terms of their demographic and socioeconomic characteristics [1,24]. Thus, we were able to represent the heterogeneity of the eastern German federal state Saxony-Anhalt.

We developed the questionnaire based on a prior literature review of existing studies regarding our subjects of interest: determinants of, reasons for, and barriers to use of preventive services. The selection of relevant instruments was additionally guided by the behavioral model of health services use by Andersen et al [20]. For the design of the questionnaire, we mainly relied on already established or frequently used instruments. The questionnaire consisted of the Short Form-12 Health Survey Version 2 of the Socio-Economic Panel (SF-12v2 of the SOEP) [25] and the German-language short form of the European Health Literacy Survey Questionnaire (HLS-EU-Q47)—the HLS-EU-Q16 [26]. In addition, several items were adapted from the Robert Koch Institute’s studies “German Health Update” (GEDA) 2014/15-EHIS [27], “The German Health Interview and Examination Survey for Adults” (DEGS) [28], the “Bertelsmann-Gesundheitsmonitor” (Bertelsmann Healthcare monitor) [29], “The National FINRISK Study” [30], “CaptureAccess” [31], and “Versichertenbefragung der Kassenärztlichen Bundesvereinigung 2020” (2020 survey of SHI insured people by The National Association of Statutory Health Insurance Physicians) [32]. Sociodemographic characteristics were recorded with items from the *Demographische Standards* (2016 edition) of the Federal Statistical Office [33]. In order to develop a questionnaire that is appropriate for the target group and did not exceed a certain length and complexity, we modified the wording or shortened some items from the original instruments.

We conducted pretesting of the questionnaire in the field with 16 participants (11 women, age range 55-84 years). In addition, we applied the method of respondent debriefing. This entailed the participants retrospectively answering open questions about the draft of the questionnaire such as regarding its comprehensibility and length [34]. The feedback was positive in general, and only minor changes were necessary (eg, verbalization of all the answer options when using Likert scales, linguistic modifications). The final questionnaire consisted of 56 questions and covered the following sections: general health status, health behavior, perceived spatial access to ambulatory health care, utilization of preventive services, health literacy and health-related information behavior, and personal characteristics. Further details about the survey sections and their operationalization are presented in Table 1.

**Table 1:** Survey sections and operationalization.

Survey section	Subtopics	Number of items
General health status	Subjective health [27], health-related quality of life [25]	5
Health behavior	Health awareness [29], physical activity [30], risk factors [28], medically diagnosed diseases^a^, social support [28], need for long-term care [28]	8
Perceived spatial access to ambulatory health care	Satisfaction with the access to medical care, existence of a general practitioner (GP), self-estimated travel time to reach a physician and the mode of transport used, forgoing medically necessary outpatient care [31]	7
Utilization of preventive services	Utilization and related behavior regarding statutory health insurance (SHI) bonus programs, dental check-ups, medical check-ups, cancer screening and vaccinations, knowledge of SHI bonus programs [28,29,32]	20
Health literacy and health-related information behavior	Health literacy [26], information channels and behavior when seeking information, need for information [29]	4
Personal characteristics	Sex [33], year of birth, partnership [28] and household [33], highest general school qualification, highest vocational qualification, employment status [33], net household income, health insurance status [28], residential region^a^, height, weight [28]	12

^a^Self-developed question.

The sex- and age-stratified random sample (women and men, each representing 50%; 20% each in the age groups 55-64, 65-74, 75-84, 85-94, and ≥95 years) was drawn from the general population via the regional population registers of the 4 municipalities. A further inclusion criterion was informed consent to participate in the study (implied consent). The targeted maximum sample size of 4000 participants could not be reached due to the demographic structure in 2 municipalities. Therefore, the gross sample consisted of 3665 people. Since long-term care home residents usually have their official residence at the nursing home in which they live, both persons from private households and residents of inpatient long-term care facilities were included in the study population.

The survey was announced in local daily newspapers and in various online media. We distributed the questionnaires in April 2021. With the questionnaire, the study participants also received a stamped, addressed return envelope allowing them to return the questionnaire directly to the Institute of Social Medicine and Health Systems Research (ISMHSR) of the Medical Faculty of the Otto von Guericke University of Magdeburg. A unique identifier (pseudonym) was assigned to all questionnaires before they were sent out. The participants were encouraged to promptly complete and return the questionnaires. As an incentive, they received a pen with the study logo. In a simultaneous mixed-mode design, participants could choose a self-administered online questionnaire as an alternative to the written postal version. LimeSurvey was used as the online survey system. The link for the online survey was provided in the accompanying information letter. The questionnaire was available in German. For the entire duration of the survey, an email contact and a telephone hotline were available for questions from the participants. The data will be analyzed using descriptive and inferential statistics (especially multivariate methods). Here, the existence of potential sociodemographic and regional differences in utilization of preventive services will be examined.

### Semistructured Interviews and Focus Group Discussions

In the qualitative part of the study, semistructured interviews were conducted with residents of Saxony-Anhalt aged from 55 years to 75 years. The interview participants were recruited in the 4 study regions covered by the survey. For this purpose, a flyer was placed in the survey envelopes for the corresponding age group. Those who were interested could contact the study team by phone or via email. Additional recruitment occurred via personal and professional networks of the study team. The initial contact was made by phone and in writing. The aim of the interviews was an exploration of the subjective perspectives and attitudes that are relevant when making a decision for or against partaking in preventive services. The perspectives and experiences of physicians will complement these findings. One focus group discussion with general practitioners (GPs) and one with medical specialists who provide the relevant preventive services were conducted for this purpose. We contacted GPs and medical specialists (gynecologists, urologists, dermatologists, and internists/gastroenterologists) in Saxony-Anhalt via personal contacts and different media (email, telephone, professional networks, medical professional associations) between the beginning of July 2021 and mid-September 2021.

For the semistructured interviews, we developed an interview topic guide. It was designed based on the findings of the literature review conducted beforehand and includes the following aspects: attitudes toward medical check-ups, cancer screening, vaccinations, and dental check-ups, as well as reasons for (non-)participation in these services and strategies for the improvement of utilization behavior. In the focus group discussions, the same topics were examined from a physician’s perspective with the aid of a discussion guide.

The interviews and focus groups took place in German. Because of the coronavirus pandemic, phone interviews were conducted. The focus group discussions were conducted using a video conference system in compliance with data protection requirements. The interviews and focus group discussions were recorded digitally. Before the interview and focus group discussions were conducted, the participants were informed about the professional background of the interviewer, the aim of the study and interviews, and data protection. In order to contextualize the insights gained from the interviews and focus groups, sociodemographic characteristics and aspects of the interview situation (eg, situational aspects and the atmosphere) were collected. The audio recordings were transcribed verbatim. The data analysis is computer-assisted using the software MAXQDA and based on Kuckartz structuring qualitative content analysis. We developed coding schemes for the interviews and focus groups. Here, we first deductively created main and subcategories based on the interview and discussion guides and in the next step inductively developed categories and subcategories on the material. We use the final coding schemes to code and analyze the entire data [35]. Depending on the nature of the data, a further analysis method may also be used.

### Stock Take and Systematic Evaluation of Informational Material

A systematic search was conducted to find digitally available informational material on preventive services on the websites of the SHIs with a dominant presence in Saxony-Anhalt and of other relevant health care actors. We chose to conduct this search with the example of colorectal cancer screening because there is obligatory informational material in the form of a brochure from the Federal Joint Committee (G-BA), which is compulsorily sent to insured people 50 years of age and older by their SHI with an invitation to participate in colorectal cancer screening [36].

We included both the SHIs with the greatest presence in Saxony-Anhalt (n=11) and other relevant health care actors (n=4) in the analysis. This also includes institutions that were explicitly referred to on the selected websites (eg, foundations). As a reference, we additionally considered the informational material from the Federal Joint Committee (G-BA). We selected the informational material from all relevant health care actors based on a defined search strategy and evaluated it systematically.

Between December 7, 2020 and July 15, 2021, we identified 37 materials on colorectal cancer screening on the websites of the aforementioned 16 health care actors. We screened various tabs and topic blocks on the websites as well as using the search field on the respective websites looking at the first hits (maximum of 100). Specific search terms were defined a priori: bowel cancer, colonoscopy, colon cancer, rectal cancer, FIT, and immunological stool test*.* Additionally, we also used the more general terms decision-making support, evidence-based information, early detection, early detection of cancer, cancer screening, prevention, and screening. Materials were included if they could be found on the websites of the health care actors or using the search field. We excluded materials that primarily described the clinical symptoms of colorectal cancer or the quality of prevention procedures (eg, colonoscopies and not early detection), which was primarily directed at people younger than 55 years of age, primarily directed at other groups of people or institutions (eg, health care providers, the press), or based first and foremost on a pictorial representation (eg, posters).

Information from a health care actor that was available in several formats (eg, the same text in the SHI members’ magazine and on the website) was only included once in the analysis.

To evaluate the material, we developed a catalog of criteria following the Guideline evidence-based health information of the German Network for Evidence-based Medicine [37]. The catalog of criteria includes the categories of transparency, text layout, content, language, frequencies, and statistical information, visualization, and accessibility.

We conducted pretesting of the catalog of criteria. Four study team members evaluated the informational material about vaccination against the human papillomavirus from 4 SHIs. After minimal adjustments for optimal applicability of the catalog of criteria (eg, language concretization), the evaluation of the material on colorectal cancer screening commenced. Two study team members independently evaluated the informational material identified on the websites. The rating scheme (from “very good quality” to “very low quality” using a 5-point Likert-scale) from Wahl and Apfelbacher [38] was adapted and modified for the purposes of our systematic evaluation. Discrepancies in the evaluation between the reviewers were discussed and resolved with a third member of the study team.

The methodology of the systematic evaluation is also to be used for informational material on other preventive services in the future.

### Ethics and Data Protection

On January 8, 2021, we received ethical approval for our study from the Ethics Committee of the University Medicine Magdeburg (200/20). Participation in the study is voluntary. The participants are informed about the aims and contents of the study as well as data protection. In the survey of residents, people consent to participation in the study by completing and returning the questionnaire (implied consent). For the semistructured interviews and focus group discussions, written, informed consent is a prerequisite for inclusion in the study. The study is conducted in strict compliance with the European Union’s General Data Protection Regulation (GDPR) and the German Federal Data Protection Act (BDSG) and in accordance with the Declaration of Helsinki [39]. For the claims data analysis, the Zi and KZV LSA transmit the data to us as completely anonymized information. The data from the survey of residents, the interviews, and focus group discussions are collected and saved pseudonymized. An independent trusted third party at the Medical Faculty of the Otto von Guericke University of Magdeburg manages data containing personally identifiable information and stores those data separately from the study data. The questionnaires for the survey of residents were sent from the trusted third party.

## Results

### Claims Data Analysis

The Zi and KZV LSA are currently preparing the requested billing data and will make them available in January 2022 for further statistical evaluation and interpretation by the study team.

### Survey of Residents

Data collection took place from April 21, 2021 to June 28, 2021. During this time period, a total of 954 people participated in the study out of the 3665 who were contacted. Of those respondents, a total of 16 people made use of the possibility to fill in the questionnaire online. The mean response proportion was 25.84%. ISMHSR staff entered the data from the questionnaires completed in written form using LimeSurvey. The data collected were checked for correctness by means of a cross-validation.

### Semistructured Interviews and Focus Group Discussions

In May 2021, 2 pilot interviews were conducted. The semistructured interviews commenced in May 2021 and were completed in July 2021. In total, we were able to recruit 18 interview participants from the 4 study regions. Two focus group discussions with physicians were conducted in October 2021. We recruited 6 GPs and 5 medical specialists across Saxony-Anhalt. Between recruitment and the actual focus groups, 2 GPs and 2 medical specialists dropped out.

### Stock Take and Systematic Evaluation of Informational Material

The systematic evaluation of digitally available informational material on colorectal cancer screening was completed in July 2021. Based on the catalog of criteria that had been developed, we identified and evaluated a total of 37 different informational materials from 16 health care actors. The results are currently being processed.

The results of the study will be published in peer-reviewed scientific journals after completion of the data collection and analysis. In addition, we are holding regular meetings with the regional cooperation partners while the study is being conducted, in order to discuss aspects of recruitment and data collection. In the final phase of the study, results shall be discussed with the purpose of a transfer from research to practice and in order to develop recommendations for improving the prevention utilization behavior in the aging population in Saxony-Anhalt.

## Discussion

### Principal Findings

This study will provide current and reliable data on the utilization of preventive services in the 55-plus age group in Saxony-Anhalt. It will provide knowledge about the determinants, reasons, and barriers associated with their use and thereby make it possible to derive prevention recommendations for the target population. Moreover, the search for information about this topic and the subsequent systematic evaluation thereof sheds light on the information available and the quality of the material. The findings can be used by actors in the social and health sectors (eg, physicians, health insurances) for target group–specific evaluation and for further development of the existing range of information and preventive services offered.

With the differentiated methodical approaches, multifaceted knowledge about the use of preventive services in old age can be generated. The mixed methods design offers the potential to gain a comprehensive understanding of the utilization of preventive services. The strengths of the methods can synergistically complement each other and compensate for limitations. The regional focus in the analysis of the Zi data makes it possible to identify the extent of regional variations in the utilization of preventive services in Saxony-Anhalt. These empirical findings can serve as the basis for deriving strategies to reduce that variation. Furthermore, the study takes into account different perspectives and views of residents and relevant stakeholders, which is essential for the identification of further potential for prevention. In the semistructured interviews and in the survey of residents, we capture the perspectives of the target group. These insights are complemented by the experiences of physicians by means of the focus group discussions. There are continual discussions with the regional cooperation partners about, for example, the feasibility of the study and the development of prevention recommendations. The results of the study are primarily relevant for the federal state of Saxony-Anhalt. They are potentially transferable to other structurally weak regions in other federal states that are strongly affected by demographic change.

### Limitations and Challenges

In 2019, the proportion of people with a migration background in Saxony-Anhalt was low, with 8% (173,100 people) compared with overall Germany (26%). Among migrants in Saxony-Anhalt, 39% had German citizenship. In the age groups of 45-65 years and ≥65 years, only 1.3% and 0.7%, respectively, had a migration background [40]. For the older population, it can be assumed that people with a migration background are mainly former contract workers from Vietnam or (late) repatriates from the former Soviet Union [41]. Therefore, migration background is not taken into account in this study. With regard to the survey of residents, there might be a social desirability bias in the respondents’ answers. Since participation in the survey of the residents was voluntary, a selective nonresponse cannot be excluded. A potential nonresponse bias will be investigated within the analysis of the survey data, taking into account sociodemographic characteristics (age and sex). For the claims data analysis, the Zi and the KZV LSA provide aggregated regional-level data. Individual-level data are not provided. As this is an ecological study, it should be taken into account that the associations found on the regional level are valid for groups of people and not for individuals. In order to underline the findings of the claims data analysis and to obtain information about individual contexts, the self-reported utilization of the preventive services being investigated is recorded in the survey of residents. Since SHI-insured persons in Germany are usually also insured for long-term care through their health insurance, the Zi data also include people in need of long-term care [9]. However, due to the aggregated nature of the data, this group cannot be shown and analyzed separately. As the interviews and focus group discussions were conducted by phone or using a video conference system, it was sometimes difficult to establish a trusting relationship with the interviewees due to the limited nonverbal communication. This challenge was addressed by conducting a phone call beforehand and thus providing the interviewees with detailed information about the study.

## Abbreviations

AiA: Autonomy in Old Age
BDSG: German Federal Data Protection Act
ERDF: European Regional Development Fund
G-BA: Federal Joint Committee
GDPR: General Data Protection Regulation
GP: general practitioner
HLS-EU-Q16: German-language short form of the European Health Literacy Survey Questionnaire
ISMHSR: Institute of Social Medicine and Health Systems Research
KVSA: Association of Statutory Health Insurance Physicians in Saxony-Anhalt (Kassenärztliche Vereinigung Sachsen-Anhalt)
KZV LSA: Association of Statutory Health Insurance Dentists in Saxony-Anhalt (Kassenzahnärztliche Vereinigung Sachsen-Anhalt)
PrimA LSA: Prevention in old-age Saxony-Anhalt (Prävention im Alter Sachsen-Anhalt)
SF-12v2: Short Form-12 Health Survey Version 2
SHI: statutory health insurance
SOEP: Socio-Economic Panel
Zi: Central Research Institute of Ambulatory Health Care in Germany (Zentralinstitut für die kassenärztliche Versorgung)
